# Spatio-temporal cluster analysis of county-based human West Nile virus incidence in the continental United States

**DOI:** 10.1186/1476-072X-8-43

**Published:** 2009-07-13

**Authors:** Ramanathan Sugumaran, Scott R Larson, John P DeGroote

**Affiliations:** 1 GeoInformatics Training, Research, Education, and Extension Center (GeoTREE), Geography Department, University of Northern Iowa, Cedar Falls, IA, USA

## Abstract

**Background:**

West Nile virus (WNV) is a vector-borne illness that can severely affect human health. After introduction on the East Coast in 1999, the virus quickly spread and became established across the continental United States. However, there have been significant variations in levels of human WNV incidence spatially and temporally. In order to quantify these variations, we used Kulldorff's spatial scan statistic and Anselin's Local Moran's I statistic to uncover spatial clustering of human WNV incidence at the county level in the continental United States from 2002–2008. These two methods were applied with varying analysis thresholds in order to evaluate sensitivity of clusters identified.

**Results:**

The spatial scan and Local Moran's I statistics revealed several consistent, important clusters or hot-spots with significant year-to-year variation. In 2002, before the pathogen had spread throughout the country, there were significant regional clusters in the upper Midwest and in Louisiana and Mississippi. The largest and most consistent area of clustering throughout the study period was in the Northern Great Plains region including large portions of Nebraska, South Dakota, and North Dakota, and significant sections of Colorado, Wyoming, and Montana. In 2006, a very strong cluster centered in southwest Idaho was prominent. Both the spatial scan statistic and the Local Moran's I statistic were sensitive to the choice of input parameters.

**Conclusion:**

Significant spatial clustering of human WNV incidence has been demonstrated in the continental United States from 2002–2008. The two techniques were not always consistent in the location and size of clusters identified. Although there was significant inter-annual variation, consistent areas of clustering, with the most persistent and evident being in the Northern Great Plains, were demonstrated. Given the wide variety of mosquito species responsible and the environmental conditions they require, further spatio-temporal clustering analyses on a regional level is warranted.

## Background

West Nile virus (WNV) is one of the most geographically widespread arboviruses in the world with cases occurring on all continents except Antarctica. In the United States it has resulted in nearly 29,000 human cases and over 1,100 deaths since its arrival in 1999 [1]. The Centers for Disease Control and Prevention (CDC) compile statistics on WNV incidence by county based on reporting from state health departments. In conjunction with the United States Geological Survey (USGS) and through their ArboNet system, this data is served in the form of maps and lists of counties with the number of WNV cases diagnosed [2]. Only a few studies have utilized this information on either a regional [3,4] or national basis [5-7]. All of these studies limited their analyses to one or up to three years of data. These studies included attempts at uncovering patterns using spatial statistics [4,5] and those investigating correlations with climatic and landscape parameters [3,4,6,7]. The present study provides a more thorough spatial (entire continental United States) and temporal (2002–2008 and cumulative during that period) description of the occurrence of WNV in humans. This study also provides statistical evidence of clustering or lack of clustering throughout the continental United States which will contribute to ongoing research by providing spatial and temporal guidance for future research.

### Spatio-temporal analysis

Knowledge of when and where outbreaks occur can lead to an understanding of the underlying causes of this potentially fatal pathogen and potential future prediction of outbreaks. There are various methods or techniques to uncover spatial patterns of disease including cluster detection, hotspot analysis, and regression models. Various spatial statistical techniques for uncovering clusters are included in some Geographic Information System (GIS) software packages as well as in various standalone programs. These programs include GeoDa, SaTScan, Crimestat, Clusterseer, and extensions for the open source statistical program R. Anselin [8] compared techniques used in four free software packages including CrimeStat, GeoDa, SaTScan, and spatial analysis packages for use in the open source R programming environment. He suggested that Kulldorff's spatial scan statistic and the Local Moran's I be used in conjunction for disease cluster analyses. Based on this recommendation we used Kulldorff's spatial scan statistic implemented in SaTScan and ArcMap's Cluster and Outlier Analysis tool which implements Anselin's Local Moran's I. Brief literature reviews for these methods are described in the following sections.

### Spatial Scan Statistic

The Kulldorff spatial scan statistic [9] is a widely implemented algorithm which allows for analysis of spatio-temporal data in order to test if diseases are clustered in space or time. The implementation of the spatial scan statistic in SaTScan has been utilized for a variety of diseases including vector-borne pathogens such as WNV. Examples of applications include those to cancer [10], diabetes [11], cardiology [12], and various infectious pathogens including malaria [13], hemorrhagic fever [14], and sexually transmitted diseases [15]. Mostashari et al. [16] developed an early warning system for WNV in New York City using SaTScan and data from a dead bird surveillance system. Similarly, Gosselin et al. [17] integrated SaTScan analyses into a comprehensive WNV surveillance system in the Quebec province of Canada. SaTScan was used to detect clusters of dead *Corvidae *locations in order to serve as an early warning system. Wimberly et al. [4] used SaTScan on county-level human WNV incidence for a seven state region in the Northern Great Plains to examine spatial clustering of human WNV incidence in 2003. They identified a significant large cluster encompassing most of North Dakota, South Dakota, and Nebraska along with parts of Montana and Wyoming. They also carried out statistical modeling comparing the WNV incidence data by county with weather, climate, and land use variables as independent variables. Human WNV incidence during 2003 was found to have the strongest relationship with long-term climatic patterns.

Although the spatial scan statistic as implemented in SaTScan is widely accepted and applied, there are acknowledged sensitivities of results to the choice of input parameters. Tango [18] pointed out that, when using SaTScan, often the most likely cluster is very large and 'swallows' neighboring regions which have non-elevated risk. Chen et al. [10] utilized SaTScan to uncover clustering of cervical cancer mortality in the US. However, they argued that the default settings for SaTScan were inappropriate, as the default setting for the maximum spatial cluster size is set at 50% of the population in the study area. They suggested investigating a variety of population thresholds and using a method for determining the most useful maximum spatial cluster size to uncover core clusters of the disease in question. Nunes [19] utilized more sophisticated geostatistical modeling in order to improve the spatial scan statistic scan window size and shape. Using too large of a scan window size can lead to the delineation of a single large cluster which consists of multiple smaller clusters with lower rates in between [20]. In using SaTScan for investigating colorectal cancer in Massachusetts, DeChello and Sheehan [21] found that a lower population threshold of 10% identified smaller, more defined areas as compared to a 25% threshold.

### Anselin's Local Moran's I

Anselin's Local Moran's I statistic was first described in 1995 [22]. The purpose of this technique is to identify clusters of features with values similar in magnitude and also to identify outliers by comparison to neighboring features and the mean of the entire population. The ability to explicitly identify spatial outliers is an advantage of the Local Moran's I statistic in relation to the spatial scan statistic. The technique is widely used and has been included in numerous commercial (ClusterSeer and ESRI's ArcGIS Spatial Statistics toolbox) and free (GeoDa and CrimeStat) software. The technique has been applied to diseases such as lymphoma [23] and other types of cancer [24-26]. Rainey et al. [23] used both the Local Moran's I and spatial scan statistic for investigating Burkitt's lymphoma in Kenya and found general agreement between the two methods in identifying clusters. They mentioned that the Local Moran's I test was less sensitive to the unique geographic features of the study area. In the Long Island area of New York, Jacquez and Greiling [25] used both Kulldorff's spatial scan statistic and Anselin's Local Moran's I test for investigating patterns of several types of cancer. They conclude that it is important to utilize more than one method when analyzing cancer mortality data. The simplicity of use [27] and ease in visualizing results [22] have been noted for the Local Moran's I. Results near edges are more suspect with the Local Moran's I as they are with many spatial statistical tests as the features near the edge will have fewer neighbors [28].

In this study we use two demonstrably effective spatial clustering techniques for identifying human WNV incidence clustering based on a record of seven years covering the continental United States. A rich set of data containing human WNV incidence data by county is available from the CDC, but until this point in time has only been incrementally investigated using spatial statistical techniques [4,5]. In this more comprehensive study we investigate patterns of WNV incidence over space and time for the continental United States. The purpose of the present study is to determine if statistically significant clustering of high or low human WNV incidence has occurred, to spatially identify any clusters in order to help focus future research, surveillance, and mosquito control, and to discuss some of the possible ecological conditions controlling the observed patterns.

## Methodology

### Data collection and processing

The number of human WNV cases by county and year were compiled from the CDC/USGS ArboNet disease map archive [2] for every year that WNV has been extant in the continental United States (1999–2008). However, only data from 2002–2008 were analyzed in this study due to the relatively low number (149) of human WNV cases in the years 1999, 2000, and 2001. We note that 2008 was included although these data were still considered provisional (last accessed March 31, 2009), by the CDC. The CDC distributes data only at a county level as the geographic level available to the public sector falls under the privacy restrictions of the Health Insurance Portability and Accountability Act (HIPAA). Although data at a more detailed geography such as zip code [29], census tract [30,31], or census block group [32] could be potentially more regionally and locally informative, county-level data are commonly used for analyses of disease occurrence at the regional [33] or countrywide [34] scale.

The number of WNV cases, for each year and a total for all years combined, was joined to the 2000 US Census shapefile of the continental United States downloaded from the National Atlas website [35]. For each year, the observed rate of human WNV incidence was calculated by dividing the number of human WNV cases by the population of the county. In addition, for each year an expected national WNV incidence rate was calculated by dividing the number of total cases for that year by the population of the continental United States. This provided an expected rate given the assumption that the WNV cases were spread evenly across all counties relative to their population. For example, in 2005 there were 3,000 reported cases of WNV in humans, and the census population of the continental United States was 281,236,122 resulting in an expected incidence rate of 0.00001067. A binary variable was assigned to each county for each year; a one was assigned when the observed rate was higher than the expected rate and a zero was assigned when the observed rate was lower than the expected rate. These binary variables were summed to calculate the number of years that a given county had higher than expected human WNV rates. A log transformed population density was calculated for graphical representation in a map.

### Spatial cluster analysis

The Kulldorff spatial scan statistic and Anselin's Local Moran's I statistic were used to analyze human WNV incidence in the US for each individual year from 2002–2008 and also for all years combined. The use of two, and specifically these two, methods has been suggested by past research [8,23,25]. It was felt that using two widely accepted clustering methodologies would provide stronger evidence of either clustering or lack of clustering of human WNV incidence. The parameterization of the two methodologies cannot be considered strictly analogous due to unique characteristics of both methodologies. However, these two methods provide a useful comparison and potentially greater evidence for WNV disease incidence patterns. For each of the methodologies, quantitative tests and visual examination were carried out to decide on appropriate input parameters for final presentation.

The Kulldorff spatial scan statistic [9] was implemented using the SaTScan 7.0.3 program [36]. The spatial scan statistic was implemented using spatial retrospective analyses to scan for areas with high rates of human WNV incidence. The analyses were carried out using the Poisson probability model, 999 Monte Carlo replications to test for significance, and allowing for overlapping clusters at different maximum spatial cluster sizes based on the percent of the population at risk. The number of WNV cases by county for each separate year was used as the case file, the county population from the 2000 census was used as the population file, and the latitude and longitude of the centroids of each county were used in the coordinates file. A range of maximum spatial cluster sizes, as suggested by Chen [10], based on population thresholds were investigated including 1, 1.5, 2, 5, 7, 10, 20, and 30% of the population at risk. The default setting of 50% used by SaTScan seemed unrealistic as indicated by other authors [10,19,21]. Climate and landscape characteristics are believed to strongly influence WNV transmission, and if a large population threshold (> 30%) was used numerous functional ecological zones controlled by climate and landscape factors would be crossed by the area within that threshold. Attempts were made to find the most meaningful population thresholds from the range tested by analyzing quantitatively and visualizing the resulting clusters. A method for evaluating homogeneity of incidence rates within clusters produced by the spatial scan statistic similar to the methodology of Chen et al. [10] was used. For each county falling in a cluster for a given year, a check was made to see if, based on the binary variable described above, the human WNV incidence rate was above the expected rate for that year. Then, for each cluster, the proportion of counties having higher than expected rates was calculated. This method was used to examine the population threshold level at which the clusters identified contained the largest average proportion of counties with high rates. These efforts were made to address the issue of large clusters containing many counties with low rates [18,20]. Chen et al. [10] suggested that this methodology should be conducted on all spatial scan statistic analyses, and that it is likely that for each disease and each different time period a different population limit would result in uncovering the "core clusters" of the disease being researched.

Anselin's Local Moran's I statistic was applied using the Cluster and Outliers Analysis tool in ESRI's ArcGIS 9.3 Spatial Statistics toolbox [37]. The human WNV incidence rates for each year and for combined years were used as variables of interest. A range of fixed Euclidean distance bands including 100, 200, 300, 400, 500, 600, 800, and 1000 km were analyzed in order to investigate potential distances at which clustering occurred. A range of thresholds was investigated as there is variation in the size of counties and population densities across the country. Additionally, the mosquito species and ecological conditions responsible for transmission vary across the country, thus a single threshold would not be suitable. An upper limit of 1000 km was used as with large distances there is greater likelihood of crossing multiple functional ecological zones. A global autocorrelation test was carried out for each year using all of the distance thresholds with the Spatial Autocorrelation (Moran's I) tool in ArcGIS Spatial Statistics toolbox. These tests were carried out to investigate at which distance thresholds the highest spatial autocorrelation for the WNV rates would be expressed.

As projected data is required for the ArcGIS spatial statistics tools, all of these analyses were carried out with projected versions of the census data with WNV rates by year. The projection used was the USA Contiguous Albers Equal Area Conic. In addition, all maps presented used this projection. All mapping was carried out using ArcGIS. After analysis of cluster homogeneity for the spatial scan statistic results, it was decided to only present map results from three of the spatial scan statistic population thresholds (1%, 2%, and 5%) as these provided the most robust clusters in that they contained higher proportions of counties with high WNV incidence. Only four of the Local Moran's distance thresholds (100, 300, 600, and 1000 km) were included in mapped results. This range eliminated the unnecessary display of a large number of maps but allowed for meaningful visual comparison to SaTScan results. These two parameterization metrics are not exactly analogous, especially due to the varying population densities across the country, but these ranges were thought to provide useful visual comparisons. The SaTScan results were overlain on the Anselin Local Moran's I results in order to provide visual evidence for areas of clustering and non-clustering. Furthermore, only three of the most significant clusters were included per spatial scan statistic percent analysis in order to provide maps which were not cluttered and difficult to interpret. This resulted in a total of nine clusters for each year being displayed.

## Results

### Rates of human WNV incidence in the US

**Figure 1:**
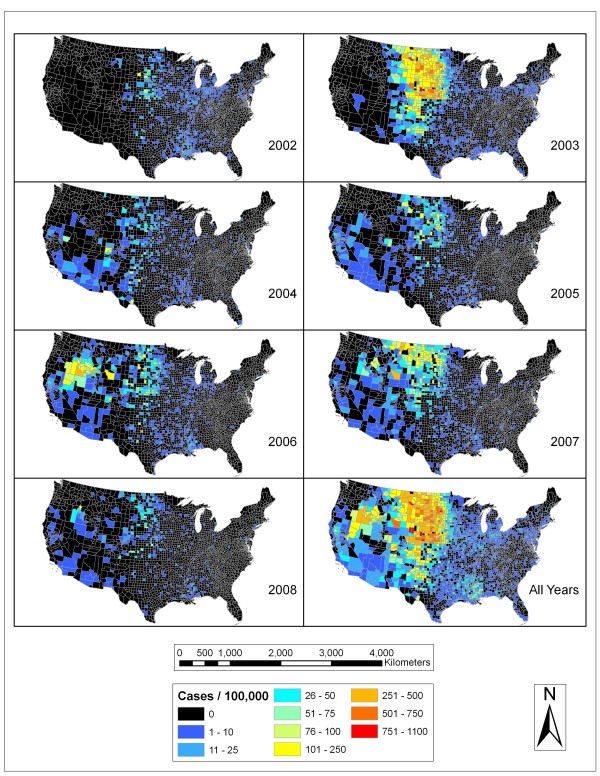
**WNV human incidence by year**. Each panel shows the number of human WNV cases per 100,000 people for a single year or for all of the years combined.

Figure 1 shows the rate of human WNV incidence for each county in the continental United States for each year in the study period. In 2002, the rates were still fairly low overall with higher rates in the upper Midwest, Northern Great Plains, and in the southeastern states of Louisiana and Mississippi. In 2003, there was a huge jump in rates, primarily in the Northern Great Plains, but spreading down into Colorado, New Mexico, and Texas. In 2004, the number of cases (2,539) was the lowest except for 2008 (1,301), but there was clear movement of the virus into California. In 2005, the case count was still fairly low (3,000) and exhibited a somewhat similar pattern to 2004 with a wider area of the Northern Great Plains standing out. In 2006, there was a grouping of high rates centered in southwest Idaho and stretching into Oregon and Nevada. There were also high rates in the Northern Great Plains region. In 2007, there were still persistent higher rates in the Northern Great Plains region with especially high rates in North Dakota and into Montana. When all of the years were combined, the Northern Great Plains area stood out as well as the area centered on southwest Idaho. There were also higher rates in eastern Colorado, northwestern Texas, Mississippi, Louisiana, central California, and Idaho.

**Figure 2:**
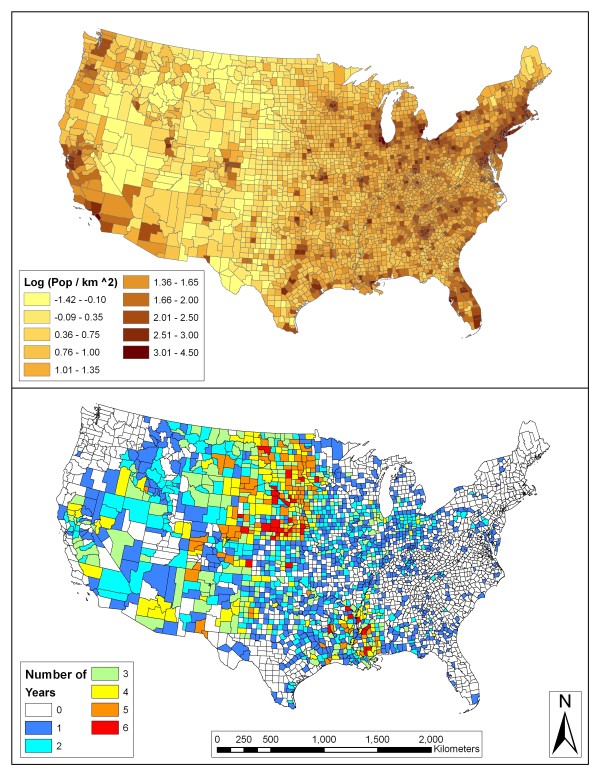
**Log-adjusted population density (people per km^2^) and US counties with higher than expected WNV human incidence**. On the top is a map of the continental United States with log-adjusted population density (people per km^2^). On the bottom is a map that displays the counties of the US with regards to how many years each county had higher than expected rates of human WNV incidence.

The top panel of Figure 2 represents population density in the continental United States. Low population densities are seen in the western half of the country including the Northern Great Plains in which there were high human WNV incidence rates. The more densely populated eastern parts of the country including the Northeast, Mid-Atlantic, and Florida had low rates of human WNV incidence as seen in Figure 1. These patterns are also seen in the map in the bottom panel of Figure 2. The Northern Great Plains region, especially counties in Nebraska, South Dakota, and North Dakota, had higher than expected rates in four or more of the seven years. Numerous counties in Louisiana and Mississippi also had higher than expected rates in more than half of the years. Several other smaller pockets of counties had several years of elevated WNV incidence. These included areas in southern Arizona, southwest Idaho, eastern Oregon, northern California, and along the front range of the Rocky Mountains in Colorado.

### Spatial scan statistic population and Moran's distance thresholds

**Figure 3:**
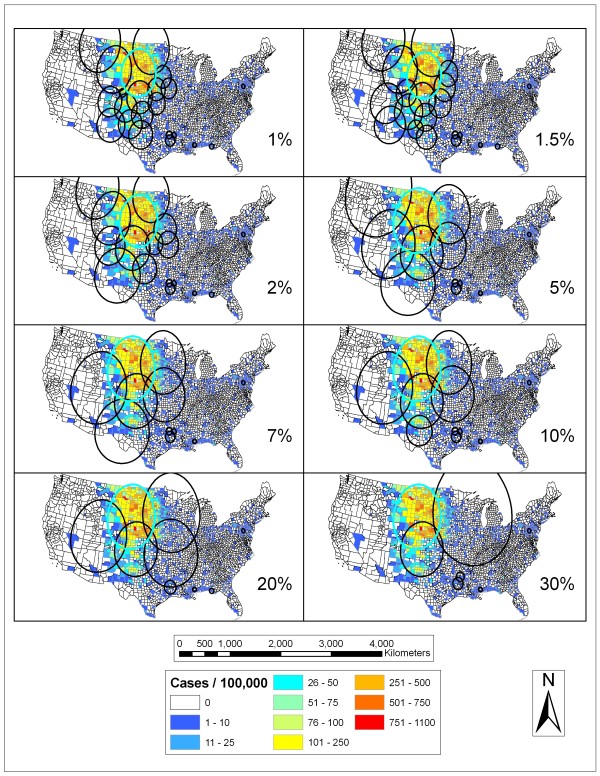
**SaTScan results for 2003 at various population thresholds**. Results of running the spatial scan statistic with varying population limits for human WNV incidence in the continental United States for 2003 are shown. Blue indicates areas with low rates of human WNV incidence and red represents areas with high rates of human WNV incidence.

In order to limit the amount of spatial scan statistic clustering results presented to a meaningful and manageable level, visual and quantitative analyses were carried out to discern useful population thresholds. Figure 3 demonstrates the results of applying various population thresholds in the SaTScan analysis of human WNV incidence for 2003. This year is used as an example to illustrate the reasoning behind limiting the population thresholds used and the number of clusters displayed. The most likely cluster, or the statistically strongest, in each map is shown in a light blue color and is similar for each of the thresholds. At population thresholds greater than 5%, a large most likely cluster covers much of the Northern Great Plains region while at lower levels these clusters are smaller and surrounded by many smaller overlapping clusters. At low population thresholds, many clusters, some of them quite small, are produced while at the larger thresholds, fewer, and sometimes larger, clusters are produced. At the higher thresholds some very large clusters detected include a large number of counties with low incidence rates. Table 1 holds the average proportion of counties within clusters for a given year/population threshold which had higher than expected incidence rates falling in those clusters. It can be seen that it is more likely that clusters at smaller population thresholds are likely to contain a higher proportion of counties with higher than expected incidence rates. Although this indicates smaller population thresholds are more useful, a potential disadvantage is that highly populated counties such as Los Angeles County, which has a population of almost 10 million (~3.5% of total), would not be considered unless the population threshold was above this level. So while small population limits have advantages, a population limit of 5% allows for the inclusion of every possible continental United States county and neighboring counties. In subsequent results, only spatial scan statistic results using the 1, 2, and 5% population thresholds are presented for the above reasons.

**Table 1:** Average proportion of counties within clusters which had higher than expected human WNV rates.

**Year**	**1%**	**2%**	**5%**	**7%**	**10%**	**20%**	**30%**
2002	*0.60*	0.56	0.49	0.49	0.42	0.42	0.41
2003	*0.66*	0.64	0.60	0.62	0.62	0.60	0.65
2004	*0.51*	0.50	0.43	0.40	0.39	0.26	0.24
2005	*0.51*	0.48	0.46	0.45	0.36	0.40	0.36
2006	*0.63*	0.57	0.54	0.52	0.46	0.41	0.57
2007	0.53	*0.55*	0.49	0.48	0.47	0.45	0.42
2008	0.43	*0.47*	0.33	0.35	0.29	0.27	0.34
All years	*0.72*	*0.72*	0.64	0.63	0.64	0.60	0.60
**Overall average**	***0.58***	**0.56**	**0.50**	**0.49**	**0.46**	**0.43**	**0.45**

For all clusters in a given year/population threshold combination, the proportion of counties within that cluster which had a higher than expected human WNV incidence rate was calculated. These proportions were then averaged for each year/population threshold and compiled in this table.

The global spatial autocorrelation test was carried out for each year/distance threshold combination to investigate the distance threshold at which there was the highest level of similarity between the human WNV incidence rates in features near each other. The highest statistical significances, as measured by Z-Scores, were for the following distance thresholds: 2002 (500 km), 2003 (800 km), 2004 (1000 km), 2005 (800 km), 2006 (1000 km), 2007 (1000 km), 2008 (800 km), and for all years combined (1000 km). In 2002, higher rates were still occurring in more densely populated eastern counties, while in later years the highest incidence was in more sparsely populated western counties thus leading to greater spatial autocorrelation at higher distance thresholds. For this study, thresholds above 1000 km were not considered as they were thought to be too large. These thresholds would encompass large portions of the country and would cross functional climatic and ecological regimes and also habitable zones of different mosquito vectors.

### Spatial clustering analysis

**Figure 4:**
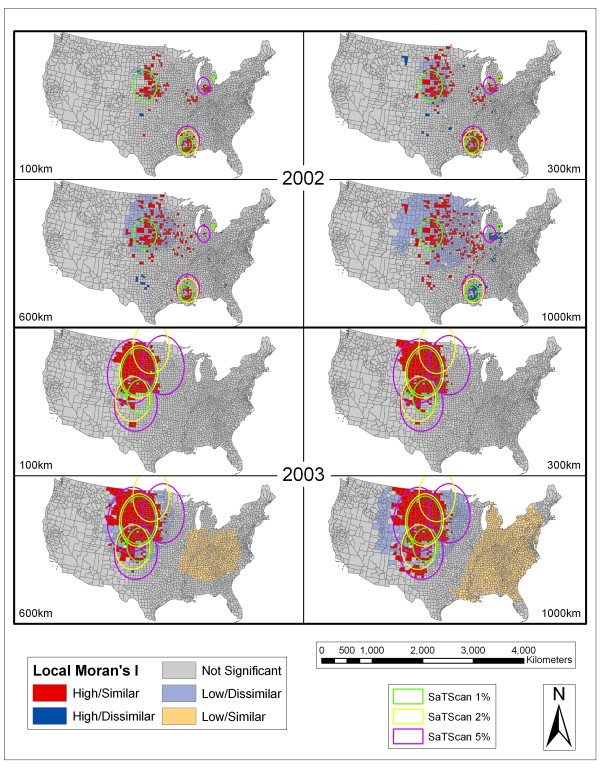
**SaTScan and Local Moran's I Analyses for 2002 and 2003**. Results of the SaTScan analyses at 1%, 2%, and 5% population thresholds overlain on the Local Moran's I analyses using 100 km, 300 km, 600 km, and 1000 km distance thresholds for 2002 and 2003. The Local Moran's I results can be interpreted as follows: high/similar are counties with high rates of human WNV incidence surrounded by counties that also show high rates of incidence, high/dissimilar are counties with high rates of human WNV incidence surrounded by counties that have low human WNV incidence, not significant are counties where there are not statistically high or low human WNV incidence levels, low/dissimilar are counties with low rates of human WNV incidence surrounded by counties that have high human WNV incidence, low/similar are counties with low rates of human WNV incidence surrounded by counties that also have low rates of incidence.

**Figure 5:**
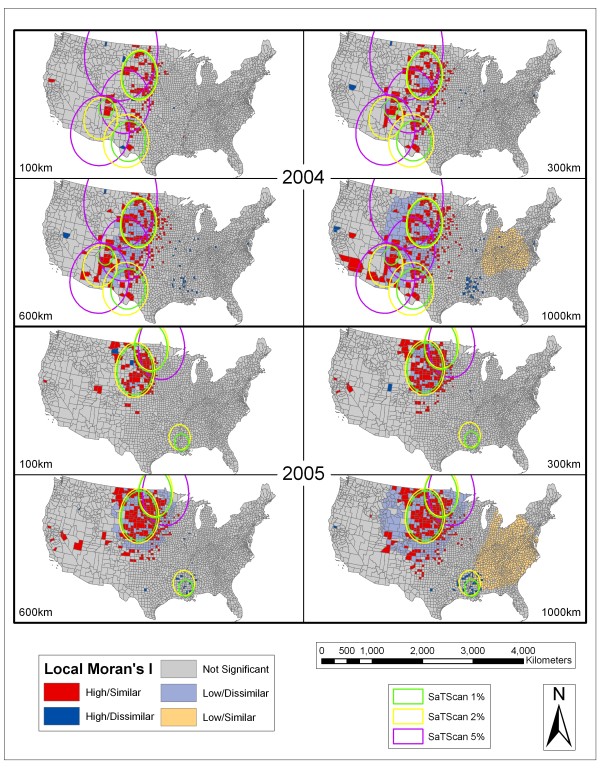
**SaTScan and Local Moran's I Analyses for 2004 and 2005**. Results of the SaTScan analyses at 1%, 2%, and 5% population thresholds overlain on the Local Moran's I analyses using 100 km, 300 km, 600 km, and 1000 km distance thresholds for 2004 and 2005. See Figure 4 for an explanation of symbology.

**Figure 6:**
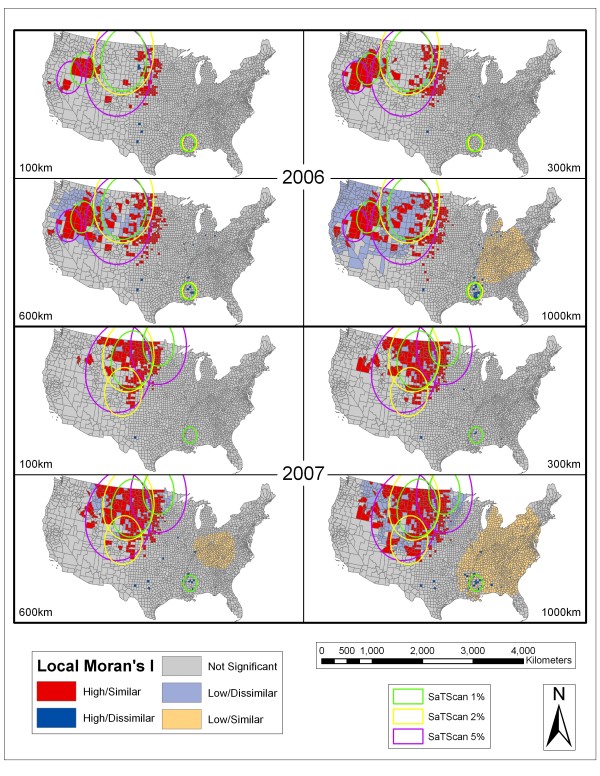
**SaTScan and Local Moran's I Analyses for 2006 and 2007**. Results of the SaTScan analyses at 1%, 2%, and 5% population thresholds overlain on the Local Moran's I analyses using 100 km, 300 km, 600 km, and 1000 km distance thresholds for 2006 and 2007. See Figure 4 for an explanation of symbology.

**Figure 7:**
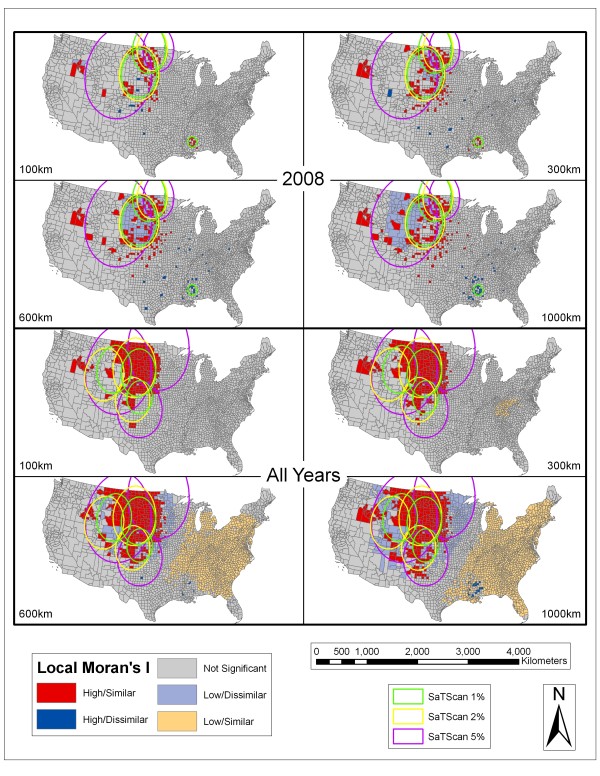
**SaTScan and Local Moran's I Analyses for 2008 and all years**. Results of the SaTScan analyses at 1%, 2%, and 5% population thresholds overlain on the Local Moran's I analyses using 100 km, 300 km, 600 km, and 1000 km distance thresholds for 2008 and all years combined. See Figure 4 for an explanation of symbology.

Figure 4, Figure 5, Figure 6, and Figure 7 depict the spatial scan statistic results based on the 1, 2, and 5% population thresholds overlain on the Local Moran's I results using 100, 300, 600, and 1000 km distance thresholds. Only three of the most significant spatial scan statistic clusters for each population threshold are displayed in these maps in order to provide interpretable maps by not over-crowding them with many small clusters. In cases where there appears to be less than three clusters for each population limit, it is because some of the significant clusters returned for the spatial scan statistic using different population thresholds were exactly the same. This more commonly occurred between the 2% and 5% population limits. In 2002, there were overlapping clusters, based on the different population thresholds, centered in Nebraska. In 2005, there were two overlapping clusters (both involving the 2% and 5% population limits). The first is centered on the Mississippi and Louisiana border. The second includes the Dakotas, Nebraska, and eastern portions of Montana and Wyoming. In 2006, there were overlapping clusters in Mississippi involving the 2% and 5% spatial scan statistic population limits. In 2007, there were again overlapping clusters in the vicinity of Mississippi involving all of the spatial scan statistic clusters. In 2008, there were overlapping clusters in Mississippi with agreement from all of the population limits displayed. The fact that the spatial scan statistic determined the exact same areas as significant clusters between different population limits shows that those specific clusters are extremely stable.

As seen in Figures 4, Figure 5, Figure 6, and Figure 7 the spatial scan statistic and the Local Moran's I results generally concur as to the areas with the greatest clustering of human WNV incidence for each year and for all of the years combined (2002–2008). In 2002, the major clusters in the Northern Great Plains, Ohio/Indiana, and Louisiana/Mississippi were captured by both methods. The spatial scan statistic clusters covered a wider area for the Ohio/Indiana and Louisiana/Mississippi clusters, while the Local Moran's I show a wider but dispersed set of clusters in the Northern Great Plains. Also the cities of Chicago and Detroit had some of the highest numbers of WNV cases in 2002. Cook County (Chicago) exhibited clustering in the Local Moran's I method except at the 100 km threshold while Wayne (Detroit) and suburban Macomb and Oakland counties in Michigan showed up as clusters at the 300 km threshold and as high outliers at the 1000 km threshold. Furthermore, the spatial scan statistic results at the 1 and 2% population thresholds showed that Oakland County was the single most likely cluster of human WNV incidence in the country in 2002. In 2003, the Local Moran's I results for all threshold distances show a large, fairly consolidated cluster covering several states in the Northern Great Plains. This area is also captured by the spatial scan statistic results at different population thresholds. However, especially at the 5% population threshold, there were peripheral clusters which contained numerous counties that the Local Moran's I statistic considered outside of clusters or in low/dissimilar areas.

In 2004, there were considerable discrepancies between the clusters shown by the two methods. The spatial scan statistic method at all of the population thresholds produced large clusters which contained a large number of counties which the Local Moran's I classified as not significant or low/dissimilar. This is reinforced by Table 1 which shows that 2004 (along with 2005 and 2008) had low proportions of counties with higher than expected WNV incidence in clusters at various population thresholds (i.e. from 0.24–0.51). The Local Moran's I clusters were generally smaller in size and ranged from the Northern Great Plains down through Colorado into Arizona, New Mexico, and Texas. In between the clustered counties were counties qualified as insignificant or low/dissimilar depending on the population threshold. The largest significant clusters in 2005 were again in the Northern Great Plains region based on both clustering methodologies. Larger peripheral clusters which contain many counties classified as insignificant by the Local Moran's I technique were shown by SaTScan. At the 1000 km distance a group of somewhat dispersed counties in the Louisiana/Mississippi area showed up as high/dissimilar. This area was also covered by the 2 and 5% spatial scan statistic clusters.

In 2006, the Local Moran's I method showed two large clustered areas centered in the Northern Great Plains and in Idaho/Oregon/Nevada. The spatial scan statistic results differed in that, although there were clusters centered on the Idaho/Oregon/Nevada area, other clusters straddled this area and the Northern Great Plains cluster shown in the Local Moran's I test. Many of the counties in the SaTScan clusters straddling these areas were classified as insignificant or low/dissimilar in the Local Moran's I method. Also, at the 1000 km distance threshold a set of counties in Louisiana/Mississippi were classified as high/dissimilar by the Local Moran's I method and also showed up as clusters in the spatial scan statistic result. The results from 2007 demonstrated characteristics similar to 2003 and 2006. There was a large area of high/similar values recognized by the Local Moran's I method in the Northern Great Plains region supported by some of the spatial scan statistic clusters. These clusters extended further west into Montana and Wyoming as compared to the 2006 clusters. There was also a smaller clustered area indicated by the Local Moran's I method centered on southwest Idaho. This area caused the 5% spatial scan statistic cluster to be pulled in that direction.

This same phenomenon was witnessed in the 5% cluster in 2008. There were few cases in 2008, and the Local Moran's I method illustrated only a few sporadic clusters in the Northern Great Plains and around southwest Idaho. Again, at the 1000 km distance threshold, there were scattered counties in the Louisiana/Mississippi area classified as high/dissimilar and these coincided spatially with spatial scan statistic clusters. When all years were combined the two most evident clusters based on the Local Moran's I method were a large area in the Northern Great Plains region stretching into Colorado and parts of Texas and also in southwest Idaho. The clusters uncovered by the spatial scan statistic reinforced the Northern Great Plains region but did not cover the southwest Idaho cluster. Again, at the 1000 km distance threshold a set of counties in Louisiana/Mississippi were qualified as high/dissimilar.

In summary, the Northern Great Plains region, with some inter-annual variation, was the most evident area of human WNV clustering throughout the study period. Both of the clustering methods demonstrated this area as consistently having clusters of high human WNV incidence. In 2006–2008, and also for the combined years, the area around southwest Idaho was consistently shown to have clusters of high values based on the Local Moran's I method. However, in 2007, 2008, and the combined years the spatial scan statistic clusters did not capture the southwest Idaho area. Counties in the southern Louisiana/Mississippi area consistently were shown as clusters based on the spatial scan statistic and were classified as high/dissimilar based on the Local Moran's I method. It should be noted that in 2003, 2004, 2005, 2006, 2007, and for the combined years at the 1000 km distance threshold there were large swaths in the eastern half of the country qualified as clusters of significantly low incidence rates. There were also a few cases where single counties were reported as being significant clusters by themselves but which were difficult to discern on the maps (e.g. Cook County, which contains Chicago, in 2002 and 2005).

## Discussion

The most consistent and largest area of clustering of human WNV incidence occurred in the Northern Great Plains. This clustering was also shown in 2003 in a SaTScan analysis by Wimberly et al. [4], carried out for a seven state region in the Northern Great Plains. We have demonstrated that significant clusters were apparent in this region for multiple years (2003, 2005, 2007, 2008, and all of the years combined) when considered in a continental United States analysis using two separate statistical clustering methodologies. Although there were consistent clusters of high and low human WNV incidence in certain areas there was spatial variation in the exact extent and location of clusters between years. Some of the potential reasons for this variation include the spread of WNV across the US, the spatial distribution of WNV vector species across regions in the US, the temporal aspect of WNV transmission, and limitations in WNV surveillance data which are discussed in the following sections. In addition, differences between the spatial scan statistic and Local Moran's I results are discussed.

### Spread of WNV across the US

The spread of human WNV incidence in the US from the East to the West as the years passed is noteworthy. In 2002, WNV had yet to reach the West Coast and clusters in both methods identified clusters centered on northern Indiana/southern Michigan, around the cities of Detroit and Chicago, Louisiana/Mississippi, and Nebraska/South Dakota with no clusters identified west of the Rocky Mountains. By 2003 (the year with the greatest number of human WNV cases), large and significant clusters occurred in the Northern Great Plains region. In 2004, the clustering was more evident in the Southwest as the virus gained a foothold in the local mosquito and bird and human populations. In the year 2006, the highest rates of human WNV incidence occurred in Idaho. By 2006, the virus was well established throughout the continental United States, and in subsequent years the Northern Great Plains region as well as the Idaho area showed the strongest clustering of high WNV incidence rates. As has been discussed in other articles [32,38], generally the year after the first significant WNV occurrence is when large outbreaks have occurred. In some WNV endemic areas in Africa roughly 90% of adults and 50% of children have developed immunity to WNV [39]. From 2002 to 2006 an evident westward movement of clusters is seen (Figure 1) with a consistent clustering pattern in the Northern Great Plains being established in that period. The mechanisms by which WNV has spread so rapidly across the continental United States is still a mystery with possible explanations including the migration of infected birds or the movement of the virus by human movement and transport of cargo and livestock.

### WNV vector distribution

The varying spatio-temporal occurrence of human WNV incidence demonstrated by our cluster analyses is at least partially driven by ecological processes that vary across regions. There are now approximately 174 recorded species of mosquitoes in the continental United States and Canada [40]. Of these species only a handful are likely to contribute significantly to the maintenance and transmission of WNV in nature [41]. This is due mainly to blood-feeding preferences, vector competence, and the differences between mosquito species' habitat requirements. *Culex pipiens *and *Culex restuans *Theobald have been considered to be the primary WNV vectors in north eastern and north central United States while *Culex tarsalis *and *Culex quinquefasciatus *are the primary vectors in much of the western United States [42]. *Culex pipiens *is considered primarily an urban mosquito while *Cx. tarsalis *is considered a rural species [43,44]. In the southeast United States, *Cx. quinquefasciatus *and *Cx. nigripalpus *are important vectors [45,46]. Other species are likely to play a minor role in virus transmission to humans (e.g. *Aedes vexans*, *Ochlerotatus trivittatus*, *Ochlerotatus triseriatus*, and other *Culex *species) [47].

The underlying reasons for the Northern Great Plains region having the highest WNV human incidence have not been fully examined or determined, but the high incidence is likely due to a unique combination of biotic and abiotic factors in this region including climatic controls. The major vector of WNV in the Northern Great Plains region is considered to be *Cx. tarsalis *[4,42]. This species is more common west of the Mississippi River according to the distribution maps of Darsie and Ward [40], and has a range that includes most of the continental United States except the majority of the New England states through the Carolinas, the southeast portion of Florida, and Michigan. The largest outbreak in the Northern Great Plains occurred in 2003 before significant immunity was built up. Bell et al. [38] demonstrated that in Grand Forks, North Dakota WNV activity was greatly reduced in 2005 as compared to 2003 even though climatic and vector conditions were very similar. However in 2005, WNV activity was greater than 2004 when there were very low temperatures and little vector or virus activity demonstrating a climatic effect [38]. In their 2002–2005 study they found only *Cx. tarsalis *to be infected with WNV. Wimberly et al. [4] demonstrated a relationship between 2003 human WNV incidence in this region with long-term climatic patterns (1971–2000) and with the percentage of the human population living in rural areas. They found a unimodal relationship with long-term May-July precipitation and a positive linear relationship with long-term May-July temperature. Their research indicated a total May-July precipitation of 200 mm would form ideal conditions for WNV amplification and transmission. A national study investigating precipitation and human WNV incidence by county found the strongest association was with annual precipitation from the preceding year [48]. Interestingly, they found the relationship to be opposite when they divided the country in two. Outbreaks of WNV in the eastern United States were more strongly associated with above-average rainfall while those in the western United States were associated with below-average rainfall although these relationships were not consistent for all years of analysis (2002–2004). While this study was correlational, the authors hypothesized that differences in the ecology of mosquito vectors contributed to this variation. They cited the hypothesis from Chase and Knight [49] that dry conditions in the previous year can cause drying of wetlands which eliminates predators of mosquitoes leading to increased populations in the following year. DeGroote et al. [32] also found human WNV incidence recorded by census block groups in Iowa in 2003, 2004, and 2005 to be significantly associated with lower annual precipitation in the preceding year. However this relationship did not hold in 2002 and 2006. Epstein and Defillipo [50] presented the argument that, historically, throughout the world droughts have been associated with WNV outbreaks. The Landesman [48] study was a rough effort at regional analysis, using the Mississippi River as a dividing line between eastern and western US. As compared to this rough methodology, the identification of clusters in this study could help to indicate more useful regions for detailed analysis of climatic associations with WNV occurrence.

In addition to climatic factors, landscape conditions, land use, and human behavior likely contribute to the increased incidence levels in the Northern Great Plains. Several studies have shown that rural areas experience disproportionately high WNV incidence including the state of Iowa [32], seven states in the Northern Great Plains [4], and nationally [7]. The national study [7], which covered 2004–2006, showed weaker associations and was probably greatly influenced by the high WNV incidence in the rural areas of the Northern Great Plains region and the area centered on southwestern Idaho which had very high incidence in 2006. It is likely that these rural agricultural regions have a high proportion of individuals who work outdoors. *Culex tarsalis *is known to be opportunistic by feeding on birds and mammals as opposed to eastern species such as *Cx. pipiens *which primarily feeds on birds [51]. This opportunistic nature means that *Cx. tarsalis *has the ability to serve early in the season as a WNV amplifying vector and later in the season as a bridge vector which passes the virus to humans. This could help to explain why WNV incidence is highest in *Cx. tarsalis*-dominated areas such as the Northern Great Plains. Reisen et al. [52] suggested that environments around farmhouses provide 'islands' of elevated vegetation which are used by birds and thus attract *Cx. tarsalis *and other mosquitoes. It is also possible that people in the Northern Great Plains spend time recreationally in concentrated areas (i.e. around water bodies) which are couched in a landscape framework in which *Cx. tarsalis *thrive and potential host bird populations congregate. Indeed, many of the irrigated areas in western states are near major rivers which also likely serve as recreation areas. *Culex tarsalis *thrives in irrigated areas and the Northern Great Plains region (especially in Nebraska, Colorado, and Wyoming) relies on irrigation for farming [53]. In Iowa, higher human WNV incidence rates were associated with rural areas and irrigated land in the western part of the state which is on the periphery of the Northern Great Plains region [32]. In a national study, Gates and Boston [7] showed a relationship between veterinary and human WNV incidence from 2004–2006 and irrigation levels. The majority of cases in these years occurred in the western United States where many states have high irrigation levels including Nebraska, Colorado, California, and Idaho. These are also states where *Cx. tarsalis *is the dominant vector. The counties in southern Idaho and the Central Valley of California experienced high WNV incidence rates and also are areas with large amounts of irrigation. The Northern Great Plains region is susceptible to drought and this is a reason why irrigation is common in this area. The interaction between climate and land use should be investigated in this region through analyses of dynamic datasets such as remotely sensed vegetation indices. While irrigation is likely an important factor in WNV transmission, especially in areas in which *Cx. tarsalis *is the dominant vector, the spatial and temporal variation in the clustering of WNV incidence cannot be explained solely by this factor as there have been high levels of WNV incidence in states with relatively little irrigation such as North Dakota and South Dakota [4]. One unexplored possible explanation for high incidence levels in the Northern Great Plains region is that a combination of vectors is playing a role in this region. In Iowa, the western half of the state had the highest human WNV incidence [32] while also having a higher proportion of *Cx. tarsalis *mosquitoes out of total *Culex *mosquitoes. However, even in western Iowa the majority of *Culex *mosquitoes and positive mosquito pools were from the *Cx. pipiens *complex which included *Cx. restuans *due to morphological similarities. According to Darsie and Ward [40], the extent of *Cx. pipiens' *range extends in a band across the northern half of the continental United States including the majority of the Northern Great Plains. Also, the western extent of *Cx. restuans' *range coincides closely with high human WNV rates in the Northern Great Plains with a narrow strip of habitat extending further west and encompassing southern Idaho which had very high rates in 2006–2008. Thus it is possible these areas are dominated by *Cx. tarsalis *with *Cx. pipiens*/*restuans *also playing a role especially in early season amplification. Based on communication with personnel from various states in the Northern Great Plains region, testing of *Cx. pipiens and Cx. restuans *for WNV was either non-existent or very limited. A further understudied aspect of WNV transmission dynamics across the Northern Great Plains region is the spatial variation in the host bird populations and how these might shift based on shifting climatic patterns. Climatic, landscape, and human behavior patterns are clearly contributing to WNV transmission dynamics in the Northern Great Plains region. However, a limited amount of spatially and temporally explicit data on the occurrence of WNV in humans, other mammals, birds, and mosquitoes has prevented a greater understanding of the dynamics in this region. The present research, as well as others [4,7,38], suggest that further study is necessary to establish why there are consistently high, but shifting, rates of human WNV incidence in this region.

Although covering less area and generally with lower rates, consistent clusters were demonstrated in other areas including counties in Louisiana/Mississippi and the Idaho area. In Louisiana and Mississippi the major vector for WNV transmission to humans is the mosquito *Cx. quinquefasciatus *[54] although other species including *Aedes aegypti*, *Aedes albopictus*, *Cx. pipiens*, *Oc. sollicitans*, *Oc. triseriatus*, and *Psorophora columbiae *were considered important in Mississippi [29]. In the southernmost area of Louisiana, *Cx. nigripalpus *is the species most commonly found to be infected with WNV [45]. In unpublished research utilizing a survey of WNV patients, it was found that 77% of the men reported hunting from October 1 through December 31, 2003 [55] perhaps implicating time of infection being during their recreational pursuits. In Mississippi, road density, stream density, slope, vegetation, and climatic conditions were used to model likelihood of WNV incidence [29]. Zip codes with higher modeled risk were more likely to have at least one case of WNV. They also concluded that WNV occurred in both rural and urban settings in Mississippi possibly implicating multiple vectors. There has been surprisingly little research published on the large WNV outbreaks in the Idaho area. However, this area is likely similar to the Northern Great Plains region in that *Cx. tarsalis *is an important vector, and irrigation might play an important role. Further research is definitely needed for this geographic area. In 2002, Detroit and Chicago had large outbreaks and clusters were demonstrated for counties containing and surrounding these cities. In comparison to some of the broad clustering patterns seen in other parts of the country, these clusters were fairly isolated and thus were not likely driven by consistent broad climatic and landscape drivers as might be considered likely across the Northern Great Plains. Rather, more local landscape and demographic (e.g. residential backyard catch basins, common in Chicago residences from certain periods, providing mosquito habitat) conditions are likely responsible as pointed out in studies in Chicago [30] and Chicago and Detroit [31]. In their local Chicago study, Ruiz et al. [30] concluded that spatially varying mosquito abatement efforts likely have significant effect on the distribution of WNV incidence. This could also be true at a national level where the level of mosquito abatement effort at a state, county, and local level varies significantly. The rural nature of the Northern Great Plains makes controlling a mosquito like *Cx. tarsalis *much more expensive per capita as compared to other places in the country. It would be difficult to gain enough data on mosquito abatement efforts to include in a national analysis.

### Temporal aspect of WNV transmission

The transmission of WNV from mosquitoes to humans also varies on a temporal scale with most cases being reported in late August and September [2]. In the southern portions of the US, mosquito species can survive year round, so you might expect to see some human cases all year long. Due to the influence of the Northern Great Plains region, the peak in overall cases across the US occurs in late summer. Human WNV incidence was found to increase with mean May-July temperature, with the percentage of irrigated cropland, and with the percentage of the human population living in rural areas in the Northern Great Plains [4]. Our dataset assembled from county case counts for each year, while informative, did not contain data at a temporal scale finer than a single year. If it were known when and where human WNV cases occurred on a weekly or monthly time-step, it would have been possible to further explore temporal aspects of WNV transmission.

### Spatial scan statistic and Local Moran's I comparison

Although the spatial scan statistic implemented in SaTScan and the Local Moran's I methods produced generally similar results, there were significant differences. The spatial scan statistic clusters often covered areas that were considered high clusters by the Local Moran's I. However, some of these clusters also contained a significant number of counties that were considered not significant or low/dissimilar in the Local Moran's I method. The Local Moran's I statistic takes into account the neighboring counties within the distance threshold to determine if there is elevated risk for a specific feature whereas the spatial scan statistic starts analyzing every individual county and then expands to include surrounding counties. The spatial scan statistic can return a single county as being statistically significant for clustering whereas the Local Moran's I would consider that county as an outlier (low/dissimilar or high/dissimilar). Both of the techniques were sensitive to the choice of parameters. At higher population thresholds, the spatial scan statistic produced large clusters containing many counties with low incidence rates. For the Local Moran's I, the number of counties and size of clusters varied depending on the threshold distance used. The larger distance thresholds brought out some counties as outliers especially in the Louisiana/Mississippi area. As population density varies dramatically across the country it is difficult to define one population or distance threshold as being ideal for either method.

### Limitations of the human WNV surveillance data

There are potential limitations in the use of WNV cases reported through the CDC ArboNet system at the county level. In general, there is underreporting of human WNV cases, because a large number of cases are asymptomatic. However, we assume that reported cases are proportionally representative of the true rate of WNV in different areas of the country. Secondly, there are likely discrepancies amongst the reporting of WNV cases by health care professionals and health departments in different states and counties. Some areas of the country lack a local health department, some health departments may not have reported all cases of human WNV incidence (it was not until 2005 that reporting of non-neuroinvasive disease caused by WNV was made mandatory), and the methodology for detection of the virus may have differed between public health departments resulting in potentially inconsistent reporting. An example of possible underreporting is Kansas, where a clear distinction is seen in 2003 WNV incidence rates along this state's border with Colorado and Nebraska (Figure 1). As known to the authors, there have been no systematic studies on WNV reporting bias thus it is difficult to account for these limitations.

The spatial and temporal resolutions of the datasets analyzed were not ideal but represented the best available for a national study. There are inherent issues when aggregating point data to various geographic boundaries. Variations in pattern of the phenomenon will be seen based on the choice of aggregation levels as is described by the modifiable areal unit problem. Thus, aggregation of human incidence data to a county level leads to a spatial representation of clustering that would differ if the aggregation level was finer or if geocoded address points of individuals with WNV were analyzed. Several studies have managed to attain data at finer spatial resolutions such as zip code [29], census tract [30,31], and census block group [32]. These studies also benefited from more precise temporal resolution than data available in this study. More detailed spatial and temporal resolution provides greater opportunity for more meaningful analysis of WNV incidence in relation to spatially variable landscape, land use, and demographic information as well as spatially and temporally variable climatic patterns. Finer scale data would also allow for the uncovering of clusters of human WNV incidence within counties and/or cities/towns and possibly reveal the potential spread of the pathogen throughout a single season. However, more detailed spatial and temporal data are unlikely to become publically available nationally due to privacy restrictions on human health data. The establishment of partnerships between academic researchers and state health departments, as seen in past research [29-32], allowing access to more detailed temporal and spatial resolution data on WNV incidence, offers the greatest promise in furthering knowledge about the pattern and dynamics of WNV transmission regionally and locally.

## Conclusion

A combination of two spatial statistics, Kulldorff's spatial scan statistic and Anselin's Local Moran's I statistic, were utilized to uncover significant spatial clusters of human WNV incidence in the continental United States at the county level. These two clustering methodologies revealed that significant clustering of human WNV incidence occurred every year from 2002–2008 and for all of those years combined. Although many smaller clusters showed up in many parts of the country, the most consistent and strongest clusters occurred in the Northern Great Plains states including large portions of Nebraska, North and South Dakota with smaller portions of eastern Montana, Wyoming, and Colorado, in Louisiana and Mississippi, and southwest Idaho. The demonstration of clusters using two separate methods provides strong evidence of their importance as WNV hot-spots. The identification of significant clusters can help focus further research efforts to uncover the underlying ecological phenomena driving the elevated human WNV incidence in these areas. Given the varying population density, ecological conditions, and dominant vectors across the country, this study could be advanced by breaking the country into more functional ecological regions and re-analyzing the clustering for these regions using these two methods. These regions should be defined based on the best available information on important vectors as well as on demography, climate, land use/cover, topography, and other landscape parameters. There is a clear need for more detailed regional and local analysis of the spatial and temporal patterns of WNV occurrence and relationships to landscape, demographic, and climatic conditions. This need is strongest in areas of consistent clustering as identified by this study such as the Northern Great Plains.

## Competing interests

The authors declare that they have no competing interests.

## Authors' contributions

RS conceived of the study, participated in its design, and helped to draft the manuscript. JPD developed Python scripts for carrying out the Local Moran's I analyses, contributed to the study design, and helped to draft the manuscript. SRL created the database of WNV incidences for the US, carried out the spatial scan statistic analyses with SaTScan, produced the figures, and helped to draft the manuscript. All authors helped to interpret results, reviewed and approved the final manuscript.
